# The Potential of Gingival Crevicular Fluid as a Tool for Molecular Diagnosis: A Systematic Review

**DOI:** 10.1155/2024/5560866

**Published:** 2024-10-15

**Authors:** María Verónica Cuevas-González, Juan Carlos Cuevas-González, León Francisco Espinosa-Cristóbal, Karla Lizette Tovar-Carrillo, Rosa Alicia Saucedo-Acuña, Alma Graciela García-Calderón, Simón Yobanny Reyes-López, Graciela Zambrano-Galván

**Affiliations:** ^1^Institute of Biomedical Sciences, Autonomous University of Ciudad Juarez, Juarez City, Chihuahua, Mexico; ^2^Research Division, Faculty of Medicine, Juarez University of the State of Durango, Durango City, Mexico

**Keywords:** a systematic review, gingival crevicular, molecular diagnosis

## Abstract

A biomarker is defined as a characteristic that is measured as an indicator of a normal biological or pathological process, a response to an exposure or intervention. Biomarkers with a diagnostic approach must identify not only the presence but also the absence of the disease with high precision, so having the biological source of the said marker is of vital importance to ensure precision and accuracy; the aim was to carry out a review of its diagnostic potential. The search strategy was carried out in three databases: PubMed, ScienceDirect, and Scopus. The keywords that were used were as follows: “gingival crevicular fluid”, “Biomarker”, and “Diagnosis”, using the Boolean operator “AND”. The filter was used at 10 years. Within the type of molecules most studied, the cytokine family was the most abundant with 25.42% of the studies, followed by metalloproteinases and proteins with 16.9% each one. Studies that included RNA-type genetic material were less frequently found. As has been demonstrated, the use of GCF as a source of biomolecules for diagnostic use has been increasing, both for oral diseases, which reflects the local conditions of the disease; it also has the ability to reflect the development of distant diseases; and this is because GCF is a blood ultrafiltrate.

## 1. Introduction

The development of periodontal disease begins with the alteration of normal oral microflora, this change is due to an oral hygiene deficiency generating a sustained state of inflammation, and all these changes stimulating the presence of defense cells such as macrophages, natural killer cells, dendritic cells, and polymorphonuclear neutrophils the function of the defense cells are neutralizing pathogenic microorganisms, controlling the inflammatory process. However, it may occur a failure in self-regulation of proinflammatory cells maintaining and evolving the inflammatory process to become chronic. This characteristic has been associated with the presence of systemic diseases because there are a probability of bacteria and/or its metabolites enter at the blood stream and development inflammation at distance [1].

Gingival crevicular fluid (GCF) is a fluid which has been recognized for its diagnostic potential since the first publications made in the late 1950s. Since this fluid is derived from the rupture of the microcapillaries of the gingival plexus, which favors the molecules founding in GCF to have the ability to reflect not only local pathological processes, referring to the supporting tissues of the tooth, but also distant pathological processes, the GCF has a composition of serum, inflammatory cells, bacteria, and their products, which are obtained by passing through the supportive tissue of the tooth [2]. As a characteristic of the periodontal tissues, the content of the crevicular fluid can influence the response of the immune system in areas remote from the oral cavity, due to bacterial metabolites that can enter to blood circulation [1].

On the other hand, a biomarker is defined as a characteristic that is measured as an indicator of a normal biological process or pathological process or as a response to an intervention or treatment [3]. The localization of the biomarker is of great relevance because it will determine the methodology for its analysis as well as the methodology to obtaining the sample and can identify systemic biomolecules found in biofluids or peripheral blood or local biomolecules identified at the specific site [4]. In recent decades, the identification of molecules in biofluids such as saliva or GCF has gained popularity, due to the great diversity of molecules that compose them that have the capacity to reflect systemic pathological processes, because these fluids are considered a blood ultrafiltration. Added to this, the easiness of obtaining the sample and the little or no invasiveness that exists at the time of sampling have made them a source of study of biomarkers not only of local diseases (oral cavity pathologies) but also of systemic diseases [5].

The recent studies focus on the diagnostic potential that this body fluid has in the diagnosis of oral diseases; however, the presence of molecules related to the development of pathologies at a systemic level has not been consolidated. Therefore, the objective of this study is to carry out a review of the diagnostic potential of GCF by identifying the main molecules related to systemic diseases and/or oral conditions. For this, a detailed search was carried out in three of the main databases to identify the original studies published in the last 10 years that focused on the study of biomolecules in the GCF as a potential diagnosis.

## 2. Materials and Methods

The protocol was registered on PROSPERO (registration number: CRD42024545672).

### 2.1. Research Question

Which are the main biomolecules identified in GCF as a potential biomarker of oral or systemic diseases?

### 2.2. Selection Criteria

#### 2.2.1. Inclusion Criteria

- Comparative descriptive, cross-sectional, longitudinal, or case-control studies.
- Studies that focus on the diagnosis of a disease or condition.
- Studies carried out in humans.

#### 2.2.2. Exclusion Criteria

- Review or systematic review type studies.
- Studies that focus on the validation of some molecular biology technique.
- Language other than English or Spanish.

### 2.3. Search Strategy

The search strategy was carried out in three databases: PubMed, ScienceDirect, and Scopus. The keywords were used: “gingival crevicular fluid”, “Biomarker”, and “Diagnosis”, using the Boolean operator “AND”. The filter was used at 10 years. The search was carried out using the keywords together and independently.

### 2.4. Study Selection

For the selection of studies, a first selection of studies was made, in which the presence of the keywords used was analyzed in title and abstract. Once the studies were selected, a second review was carried out, in which the presence of the variables to be studied was identified. The evaluations of the studies were carried out by two evaluators independently; in case of discrepancy, a third evaluator participated.

### 2.5. Risk of Bias

The analysis of risk of bias was performed using the JBI critical appraisal tool for each type of study design [6].

### 2.6. Data Extraction and Analysis

The information extracted from each study was as follows: author, year, disease and/or alteration under study, type of molecule studied, methodology used to study the said molecules, and main findings demonstrated.

The information was entered into a database, SPSS V.22; only a descriptive analysis of the data was carried out, reporting the mode for the qualitative variables.

## 3. Results

### 3.1. Search Strategy

**Figure 1:**
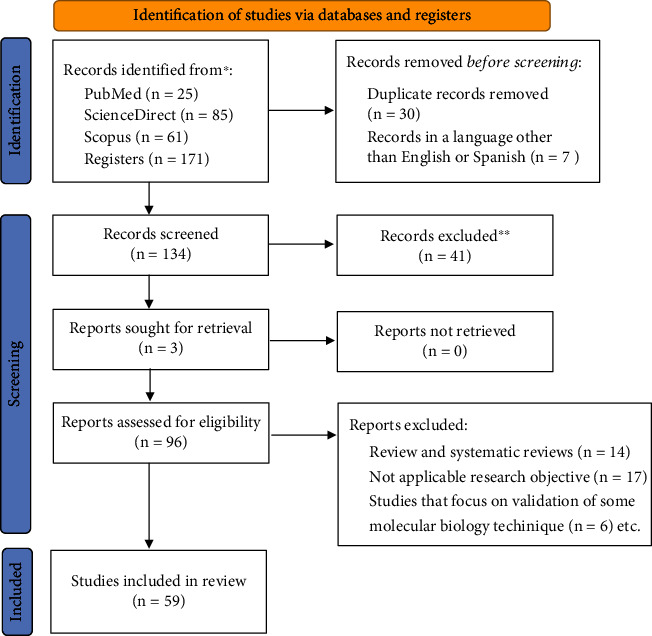
PRISMA 2020 Flow Diagram.

The search strategy yielded a total of 171 studies in the three databases (PubMed, Scopus, and Science Direct), of which 96 were selected as potential studies. When carrying out the extensive review, 59 studies were selected to be included in the present work (Figure 1).

### 3.2. Data Analysis

Of the total number of studies included, the oral disease was the most common with a 49.5% of which 29 corresponded to periodontal disease and five studies to other oral diseases or conditions. The studies that focused on the relation between periodontal disease and obesity were 13.4% (*n* = 8), followed by periodontal disease and diabetes with 8.4% (*n* = 5).

Within the type of molecules most studied, the cytokine family was the most abundant with 25.42% of the studies, followed by metalloproteinases and proteins with 16.9% each one. The studies that included RNA-type genetic material were less frequently founded. When analyzing the molecular strategies that were used for the identification of these molecules, the enzyme-linked immunosorbent assay (ELISA) in 34 studies was used.

**Figure 2:**
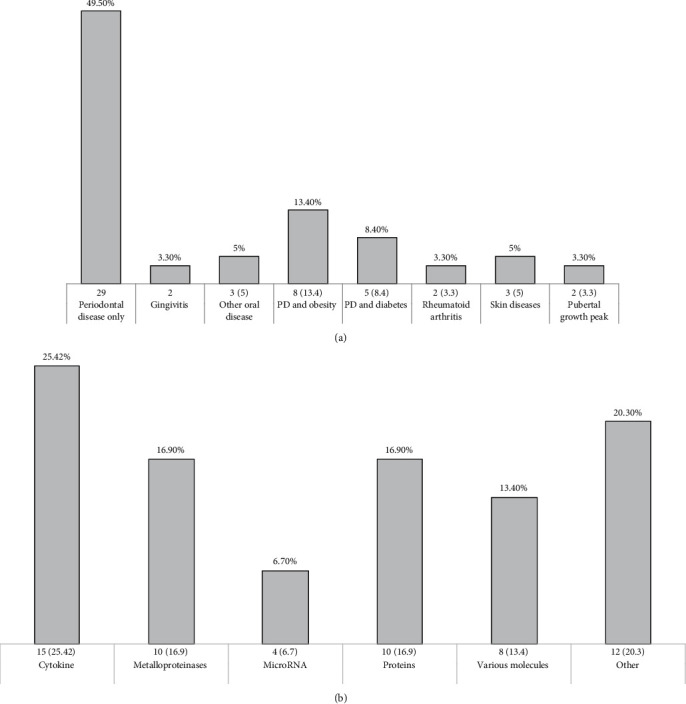
Descriptive analysis: (a) frequency of diseases studied through the gingival crevicular fluid and (b) main molecules studied in the gingival crevicular fluid.

Finally, according to the results shown, 57 from 60 the studies mentioned that GCF has diagnostic potential and only three studied mentioned having found no differences between the groups (Tables 1 and 2 and Figure 2).

### 3.3. Risk of Bias

**Figure 3:**
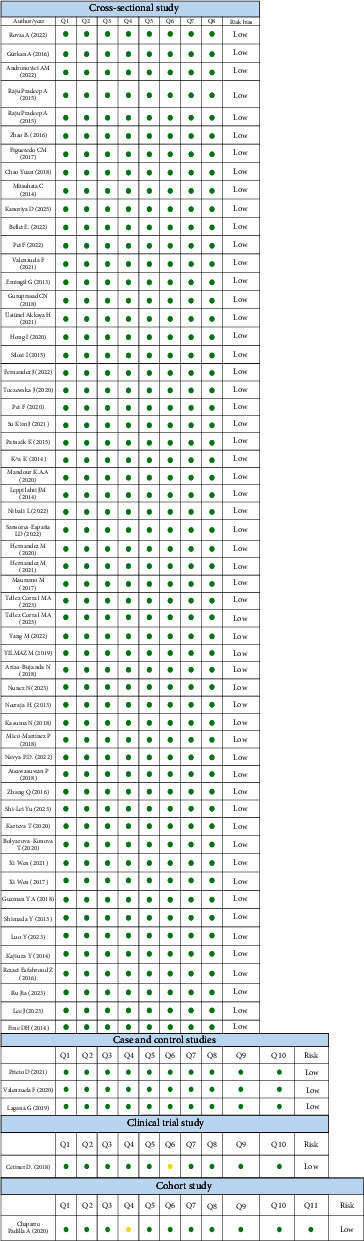
Risk of bias.

The analysis of risk of bias was performed using the JBI critical appraisal tool for cross-sectional study, cohort study, and case and control study; for each one, we identify a low risk of bias (Figure 3).

## 4. Discussion

The diagnostic potential of GCF has been increasing over time, encompassing not only diseases of the oral cavity but also systemic diseases. In the present study, it was identified that a large part of the studies involves the diagnosis of systemic diseases like metabolic syndromes, obesity, and dermatological alterations, evidencing that this type of body fluid has a large array of molecules that represent the general condition of the individual. However, the presence of biomolecules has been closely related to the early diagnosis or progression of periodontal disease. This disease is characterized by the presence of sustained inflammation of tooth-supporting tissue, thanks to the presence of proinflammatory cytokines, together with the action of metalloproteinases, and generated tissue destruction, so the patient presents not only inflammation and gingival bleeding but also gingival recession, root exposure and, in severe cases, mobility and subsequent loss tooth because of destruction of alveolar bone [66].

### 4.1. Cytokines

Cytokines are soluble proteins secreted by cells of the immune system whose main function is to regulate the defense system, either activating or repressing it through pro- or anti-inflammatory cytokines. The greatest clinical relevance of cytokine is related to immune cell differentiation, inflammation, angiogenesis, tumorigenesis, neurobiology, viral pathogenesis, atherosclerosis, cancer, and aging [67]. The overexpression of these proteins in body fluids is an excellent biomarker of the presence of inflammatory processes or their control, so it is not surprising that a large percentage of studies included in this work will focus on the differential expression of these molecules in different pathologies [68], and this was demonstrated by Sansores-España et al. in a very interesting work in which the relationship between periodontal disease and the development of Alzheimer's Disease was demonstrated through the expression of cytokines in GCF, showing that the presence of IL-1*β*, IL-6, IL-17, and TNF-*α* are considered a risk for the progression of Alzheimer's Disease [39]. On the other hand, Andronovici et al. mention that periodontal disease has a direct impact on the development of chronic kidney disease, when they evaluated the presence of TNF-*α*, IL-1*β*, and MMP-8 in patients with hemodialysis and periodontal disease and identified significant differences in TNF-*α* and MMP-8 among its groups of studies that were included [9]. Finally, adipose tissue is considered an endocrine organ capable of producing adipokines [69]. Çetiner et al. analyzed the relationship of obesity with the presence of periodontal disease through the expression of adipocytokine displaying that the presence of visfatin and IL-6 was higher in obese subjects [17]. All above corroborates that molecules related to oral pathologies and the development or progression of different systemic disorders can be identified in the GCF. A possible explanation is that in subjects with metabolic deregulations, they are usually related to periodontal problems [70].

### 4.2. Metalloproteinases

The most studied molecules were metalloproteinases, which are a family of proteolytic enzymes whose main function is the degradation of proteins of the extracellular matrix. It is worth mentioning that most of these enzymes are not found constitutively in tissues and their expression is induced by exogenous signals such as cytokines, growth factors, or altered cell-matrix and cell-cell contacts; the half-life of the expression of its mRNA can be regulated in the same way by growth factors and cytokines [65, 71]. A deregulation in the expression or in the activity of metalloproteinases can result in the development of several diseases ranging from cardiovascular alterations to inflammatory diseases and in the development of malignant neoplasms [72]. In the GCF, it is logical to find this molecule since it is related to the destruction of the tooth-supporting tissues as a result of the chronic inflammatory process that occurs in a local way; however, the relationship of this group of molecules with other alterations has been studied at a systemic level. Laganà et al. analyzed the relationship of metalloproteinases with Marfan syndrome, identifying the presence of MMP-9 and MMP-2 in GCF, which may be related both to the maintenance of inflammatory states and to the active remodeling processes of dental tissues [24]. On the other hand, Silosi et al. studied the relationship between periodontal disease and rheumatoid arthritis, identifying the differential expression of MMP-9 in subjects with rheumatoid arthritis and chronic periodontal disease [29]. Finally, the differential study of these molecules has also been related to dermatological alterations such as rosacea. Fernández et al. analyzed the presence of MM-P-2 Y 9 as a potential diagnosis, regardless of the patient's periodontal status, demonstrating that MMP-9 have a differential expression in subjects with rosacea, suggesting that the systemic inflammatory state generated can be identified in the GCF, independently of the status of the periodontium [30]. The latter shows us the great diagnostic value of the GCF.

### 4.3. MicroRNAs

MiRNAs are short noncoding RNAs that regulate gene expression by binding to specific mRNA targets and promoting their degradation and/or translational inhibition which results in a decrease or overproduction of proteins that can generate alterations in cellular and/or tissue homeostasis [73]. Another disease that stood out in our study was the relationship between obesity and periodontal disease, due to the secretion of cytokines that maintain an inflammatory state, as demonstrated by Pradeep et al. when studying the presence of vaspin, a molecule produced by adipose tissue, in subjects with periodontal disease with and without obesity, showing a differential expression between groups [10]. On the other hand, regulatory molecules such as microRNAs have also been studied as support in the diagnosis of periodontal disease; one of them is miRNA-1226, which is related to CALR overexpression, protein linked to a decrease in mineralization [50] following the same molecular line. Atsawasuwan et al. identified miRNA-29 overexpression in subjects with orthodontic treatment; this microRNA is related to the activation of the activity of the osteoclasts [52].

One of the objectives of the study of new biomarkers is the feasibility and viability of its identification avoiding complex molecular strategies to handle on a large scale or expensive, and the ELISA immunoassay is one of the most used strategies for this purpose that allows quantitative information on the protein expression [74].

All the previous evidence corroborates the potential that GFC has as a source of biomarkers for different diseases; however, we detected that most studies are in the exploratory phase, making it difficult to establish these molecules as true biomarkers. The clinical validation of these molecules to be able to establish them as biomarkers includes the analysis of expression in larger populations and double-blind studies, validating not only the expression of the molecule but also the methodology used for its identification [75]. Although some studies failed to significantly demonstrate the diagnostic potential of certain molecules, this may be due to several factors, the varying conditions of the oral cavity, which can vary widely between subjects. Another possible explanation would be the number of subjects studied, as well as the methodology used; however, these studies create bases for future guidelines in the analysis of GCF.

## 5. Conclusions

According to what was observed in this study, we can conclude that the target molecules for identification in the GCF are cytokines followed by metalloproteinases. These molecules not only represent the local inflammatory state, but they also have the ability to reflect alterations located at a distance, as well as relating to the risk of developing systemic conditions.

As it has been demonstrated, the use of GCF as a source of biomolecules for diagnostic use has been increasing; however, we consider that it is time to continue these studies by various research groups with the aim of analyzing the differential expression in larger populations and under different controls to be able to establish them as true biomarkers.

## Figures and Tables

**Table 1:** Main findings collected through the study.

**Author (year)**	**Type of disease**	**Type of molecule**	**Specific molecule**	**Molecular technique used for diagnosis**	**Main results**	**Ref**
Rovas et al. (2022)	Periodontal disease	MicroRNA	miR-140-3p, miR-145-5p, miR-146a-5p, and miR-195-5p	RT-PCR	miR-146a-5p in GCF were strongly associated with the presence and severity of periodontal disease	[7]
Gürkan et al. (2016)	Metabolic syndrome and gingivitis	Chemokine and cytokine	MCP-1, RANTES, and MIF	ELISA	RANTES level has significantly elevated in MetS patients with gingivitis	[8]
Andronovici et al. (2022)	Chronic kidney disease and periodontitis	Cytokine, metalloproteinases, and glycoprotein	TNF-*α*, IL-6, and MMP-8	ELISA	The level of TNF-*α*, IL-1 beta, and MMP-8 points out the increased inflammatory status of the dialysis patient with periodontal disease	[9]
Pradeep et al. (2016)	Obesity and periodontitis	Adipokine	Vaspin	ELISA	Our results indicate that vaspin levels were positively correlated with body mass index, clinical attachment level, and periodontal disease	[10]
Pradeep et al. (2016)	Obesity and periodontitis	Glycoprotein	Lipocalin-2	ELISA	The levels of Lcn2 were notably higher in obese subjects compared to nonobese subjects	[11]
Zhao et al. (2016)	Children obesity and periodontitis	Cytokine	TNF-*α*	ELISA	TNF-*α* is significantly elevated in gingival crevicular fluid of obese children	[12]
Figueredo et al. (2017)	Type 2 diabetes and periodontitis	Cell-derived microparticles	Panel	Flow cytometry	Neutrophil and platelet-derived microparticles in gingival crevicular fluid collected from patients having both severe periodontitis and type 2 diabetes	[13]
Yuan, Liu, and Zheng (2018)	Periodontal disease	Metalloproteinases	aMMP-8	ELISA	Demonstrate a clear positive correlation between aMMP-8 levels and clinical periodontal index, including periodontal disease or bleeding index parameters	[14]
Mitsuhata et al. (2014)	Periodontal disease	Enzymes	*N*-Bezyoyl-DL-arginine-2-naphthylamide (BANA) and aspartate aminotransferase (AST)	BANA-Zyme test strip and PTM assay	AST levels can be used to assess periodontal conditions objectively according to the degree of inflammation	[15]
Fine et al. (2014)	Localized aggressive periodontitis	Cytokine	Macrophage inflammatory protein-1a	PCR and multiplex assay	MIP-1a in GCF can identify sites susceptible to bleeding index	[16]
Çetiner et al. (2018)	Obesity and periodontitis	Cytokine	Visfatin, IL-6, and TNF-*α*	ELISA	Visfatin and IL-6 were higher in obese individuals compared to their nonobese controls and in individuals with GCP compared to their healthy periodontal controls	[17]
Kanoriya et al. (2017)	Obesity and periodontitis	Adipokine and hormone	Retinol-binding protein 4 (RBP4) and leptin	ELISA	The levels of RBP4 and leptin were notably higher in obese patients compared to nonobese patients	[18]
Prieto et al. (2021)	Rheumatoid arthritis	Nontyrosine kinase	NRP-1	ELISA	Rheumatoid arthritis patients had significantly higher levels of sNRP-1 in GCF compared with healthy controls	[19]
Bellei et al. (2022)	Severe periodontal disease	Proteomic	Panel	Liquid chromatography with tandem mass spectrometry (LC-MS/MS)	Seven of the upregulated proteins (A2M, SERPINB1, SERPINA3, HP, HPX, GAPDH, and CALR) and 10 of the downregulated proteins (KRT13, KRT14, KRT16, KRT19, KRT4, KRT6A, ENO1, GC, C3, and SERPINA1)	[20]
Pei et al. (2022)	Gingival recession in orthodontic	Proteomic		LC-MS/MS	26 potential biomarker candidates involved in GR during orthodontic treatment, including GUSB, NPM1, VASP, TLN1, FERMT3, PTPN6, ACTN1, FN1, KPNB1, IQGAP1, SEC61G, RPL35, AGRN, RALA, and MYO1C	[21]
Valenzuela et al. (2021)	Psoriasis	Cytokine and adhesion molecule	IL-18 and E-selectin	Multiplex bead–based immunoassay	The GCF levels of IL-18 and E-selectin were associated with moderate to severe psoriasis based on clinical and histopathological diagnoses	[22]
Valenzuela et al. (2020)	Moderate/severe atopic dermatitis	Protease	Panel	Multiplex bead immunoassay	Single use of MMP8 allowed for the correct identification of both conditions (AD and healthy subjects), indicating that the GCF could serve as a novel and noninvasive source for potential diagnostic biomarkers of moderate/severe atopic dermatitis	[23]
Laganà et al. (2019)	Marfan syndrome	Metalloproteinases	Panel	Zymography	In GCF from Marfan patients, a very high pro-MMP-9 levels were observed; likewise, when MMP-2 activity was identified, this was more intense in the GCF from Marfan patients	[24]
Emingil et al. (2013)	Generalized aggressive periodontitis	Metalloproteinases	MMP-8 and TIMP-1	PCR genotyping	MMP-8-799 C/T, TIMP-1 372 T/C, and ∗429 T/G gene polymorphisms in males are associated with GAgP	[25]
Guruprasad and Pradeep (2018)	Obesity and periodontitis	Cytokine	IL-34	ELISA	The results showed that mean levels IL-34 in GCF were increased in patients who were obese and had periodontitis	[26]
Akkaya et al. (2022)	Periodontal health, gingivitis and Stage III Grade C periodontitis	Enzymes and cytokine	Galectin-3, PAD4, and TNF-*α*	ELISA	Elevated GCF levels of galectin-3 and PAD4 in periodontitis compared with healthy controls suggest that these molecules play important roles in periodontitis pathogenesis	[27]
Hong et al. (2020)	Gingivitis	Proteases	Cystatin C, MPO, and MMP-9	ELISA	MMP-8 and MPO levels are suitable for diagnosing gingivitis	[28]
Silosi et al. (2015)	Rheumatoid arthritis and periodontal disease	Metalloproteinases	MMP-9	ELISA	Elevated serum and GCF MMP-9 levels were observed for chronic periodontitis, rheumatoid arthritis, and associated rheumatoid arthritis-chronic periodontitis	[29]
Fernández et al. (2022)	Rosacea	Metalloproteinases	MMP-2 and MMP-9	Multiplex bead immunoassay	Reported higher levels of MMP-9 in the GCF of patients with rosacea compared to systemically healthy controls	[30]
Toczewska et al. (2020)	Periodontal disease	Nitrosative stress	Nitric oxide, peroxynitrite, and *S*-nitrosothiols)	BCA protein assay	Nitrosative stress markers did not correlate significantly with the parameters of periodontium clinical condition	[31]
Pei et al. (2020)	General chronic periodontitis	Metabonomic	Panel	16S rRNA amplicon sequencing	Successfully screened 17 differential metabolites in GCF samples by GC-MS possibly separating patients with GCP from healthy controls (glycine-d5, *N*-carbamylglutamate 2, fructose 1, 2-butyne-1,4-diol, 5-dihydrocortisol 3, *N*-acetyl-beta-*D*-mannosamine 1, 4-hydroxyphenylacetic acid, citramalic acid, uracil, beta-glutamic acid 1, monoolein, methylmalonic acid, thymidine 3, octadecanol, 1-monopalmitin, *O*-phosphoserine 1, lactamide 2)	[32]
Kim et al. (2021)	Periodontal disease	Proteomic	Panel	LC-MS/MS	It was determined that Gal-10 protein levels were high in GCF from patients with periodontitis	[33]
Patnaik et al. (2017)	Chronic periodontitis and type 2 diabetes mellitus	Cytokine	Human chemerin	ELISA	Increase in the GCF and tear-fluid levels of chemerin from healthy to CP to type 2 DM with CP	[34]
Ka et al. (2014)	Gingivitis	Hormone and cytokine	Osteocalcine and TNF-*α*	ELISA	Found a statistically significant negative association between plasma ucOC and GCF TNF-*α* level in 8–10-year-old Caucasian children	[35]
Mandour, Tawfeek, and Montasser (2021)	Root resorption	Cytokines and protein	IL-1*α*, IL-1*β*, and dentin sialo phosphoprotein	ELISA	The levels of IL1-ra and DSPP that were detected in the orthodontic and pediatric groups indicate a possible association of the biomarkers with OIRR	[36]
Leppilahti et al. (2014)	Chronic periodontitis	Metalloproteinases and myeloperoxidase	CXCL5, MPO, MMP-8, and TIMP-1	ELISA	MPO and collagenolytic MMPs represent highly discriminatory biomarkers for site-specific diagnosis of periodontitis	[37]
Nibali et al. (2022)	Metabolic syndrome	Cytokine	Panel	Cytokine array	Aggrecan is present at relatively high levels in the GCF and that it may be inversely correlated with the severity of periodontitis	[38]
Sansores-España et al. (2022)	Alzheimer's disease	Cytokine	Panel	Multiplex immunoassay and ELISA	The data allow us to suggest that in patients with AD, there is a higher burden of *Porphyromonas gingivalis*, clinical attachment level loss, higher bleeding on probing and plaque index, and higher levels of proinflammatory and probone resorptive mediators in the GCF	[39]
Hernández et al. (2020)	Periodontal disease	Metalloproteinase	MMP-8	ELISA	MMP-8 had the highest accuracy to discriminate between healthy and periodontitis sites, while MMP-8 demonstrated the best diagnostic precision in the detection of mild from severe periodontitis sites	[40]
Hernández et al. (2021)	Mild and severe periodontitis	Metalloproteinase and cytokine	MMP-8, TRAP-5, and OPG	Immunofluorometric method	MMP-8, TRAP-5, and OPG in GCF exert a high diagnostic performance in periodontitis, discriminating between incipient/early and more advanced levels of the disease	[41]
Mauramo et al. (2018)	Periodontal disease	Metalloproteinase	MMP-8	Immunofluorometric assay	Periodontitis was observed to be statistically significantly more prevalent in subjects with higher levels of MMP-8 in GCF	[42]
Téllez Corral et al. (2023)	Obstructive sleep apnea	Cytokines	IL-1*β*, IL-6, IL-17A, IL-33, and TNF-*α*	Multiplex bead immunoassays	Higher prevalence of Stage III periodontitis and severe OSA, as well as higher levels of periodontal parameters (PI and BOP), and expression of IL-1*β* and IL-6 in saliva and IL-6, IL-17A, and IL-33 in GCF	[43]
Yang, Soh, and Heo (2022)	Severe periodontitis (Stage III/IV)	Hydrolase	Acidic mammalian chitinase	Western blot and ELISA	Concentration of AMCase was significantly increased in the severe periodontitis group	[44]
Yilmaz et al. (2019)	Generalized aggressive and chronic periodontitis	Metalloproteinase and bacteria	MMP-3 panel bacteria	16S rRNA amplicon and ELISA sequencing	MMP-3 levels in patients with AgP are statistically higher compared to the healthy sites. Unexpectedly, even the healthy sites in generalized aggressive periodontitis patients showed higher levels of pathogens, especially red complex bacteria and *Fusobacterium nucleatum*	[45]
Arias-Bujanda et al. (2018)	Chronic periodontitis	Cytokines	Panel	Multiplexed immunoassays	IL1alpha, IL1beta, and IL17A, and their ratios with IL2, are excellent diagnostic biomarkers in GCF for distinguishing periodontitis patients from periodontally healthy individuals	[46]
Nunez et al. (2023)	Apical periodontitis	Cytokines	Panel	Multiplexed bead immunoassays	TNF-*α* and IL1-b were elevated in most teeth with apical periodontitis compared to healthy control teeth	[47]
Gokhale et al. (2014)	Chronic periodontitis and type 2 diabetes mellitus	Adipokine	Resistin	ELISA	GCF resistin levels correlate with periodontitis and DM, individually and simultaneously	[48]
Kasuma et al. (2018)	Periodontal disease	Metalloproteinase	MMP-8	ELISA	The mean MMP-8 concentrations are higher in mild periodontitis patients than it is in mild gingivitis and healthy individuals	[49]
Micó-Martínez et al. (2018)	Chronic periodontitis	MicroRNA	Panel	RT-PCR	miR-1126 can provide clinicians another tool to identify susceptible patients and may add relevant information to common clinical parameters used for diagnosis and prognosis of periodontitis	[50]
Navya et al. (2022)	Diabetes mellitus and periodontitis	Interleukine	Suppression of tumorigenicity 2	ELISA	Results showed that the mean value of ST2 in GCF was significantly high in periodontitis diabetes mellitus group	[51]
Atsawasuwan et al. (2018)	Orthodontic tooth movement	MicroRNA and exosome	Panel	Western blot and PCR	miRNA-29 family expression in GCF were associated with osteoclast activity during the tooth movement	[52]
Zhang et al. (2016)	Periodontal disease	Cytokine	IL-6, IL-10, TNF-a, CRP, and ALP	ELISA	IL-6, IL-10, TNF-a, CRP, and ALP levels in the GCF of the tooth site correlate with the severity of periodontal destruction	[53]
Yu (2023)	Chronic periodontitis	MicroRNA	miR-200 family	RT-PCR	miR-200a, miR-200b, and miR-200c demonstrated significant upregulation in the GCF of CP patients compared to healthy controls	[54]
Karteva (2020)	Apical periodontitis	Metalloproteinase	aMMP-8	ELISA	The levels of activation of MMP-8 in GCF do not increase to pathologically elevated levels in teeth with AAP	[55]
Bolyarova-Konova et al. (2020)	Periodontal disease	Cytokine	IL-1*β*	ELISA	Found significantly higher mean rank salivary and GCF concentrations of IL-1*β* in patients with overt periodontitis as well as with gingivitis compared to healthy subjects	[56]
Wen and Gu (2021)	Pubertal growth peak	Glycoprotein	Transferrin and vitamin D binding protein	ELISA	The percentage of TF in GCF of pubertal subjects was significantly higher than prepubertal and postpubertal subjects, both in maxilla and mandible	[57]
Wen et al. (2018)	Pubertal growth peak	Proteomic	Panel	LC-MS/MS	Vitamin D binding protein and transferrin levels in pubertal subjects were significantly higher than those in postpubertal subjects	[58]
Guzman et al. (2018)	Chronic periodontitis	Proteomic	Panel	MS	Identify azurocidin, lysozyme C, myosin 9, and smooth muscle actin as candidate biomarkers which can aide in the determination of clinical endpoints for CP	[59]
Shimada et al. (2013)	Periodontal disease	Panel	Panel	Multiplex immunoassays	Detected 26 biomarkers in GCF and clarified the presence and quantity of *P. gingivalis* from the same sites using a multiplex bead immunoassay	[60]
Luo, Ding, and Chen (2023)	Chronic periodontitis	Cytokine	IL-31 and IL-34	ELISA	IL-31 and IL-34 play essential roles in the detection and treatment response of CP	[61]
Kajiura et al. (2014)	Periodontitis and type 2 diabetes	Protein	Glycated albumin	ELISA	The present study shows the possibility of predicting DM-P using GA, a DM marker, and calprotectin, an inflammatory marker, in GCF	[62]
Esfahrood et al. (2016)	Chronic periodontitis	Cytokine	IL-8	ELISA	No significant increase was found in the level of IL-18 in saliva and GCF of chronic periodontitis compared with healthy subjects	[63]
Jia et al. (2023)	Obesity and periodontitis	Cytokine	Adipokines panel	ELISA	Obese patients have worse periodontal conditions and significantly high levels of inflammatory cytokines and adipokines expression	[64]
Lee et al. (2023)	Periodontal disease	Immune system	DEFA-1	Western blot and ELISA	ELISA quantitative analysis confirmed that DEFA-1 distinguished between the healthy condition and periodontitis	[65]

**Table 2:** Descriptive analysis of the studies.

**Characteristic of study**	**n** ** (%)**
Year	
2013	3 (5)
2014	4 (6.7)
2015	5 (8.4)
2016	3 (5)
2017	4 (6.7)
2018	8 (13.4)
2019	2 (3.3)
2020	9 (15.2)
2021	6 (10.1)
2022	9 (15.2)
2023	6 (10.1)
Design of study	
Cross-sectional	55 (93.3)
Case and control	3 (5)
Clinical assay	1 (1.6)
Studies by database	
PubMed	11 (18.6)
ScienceDirect	25 (42.37)
Scopus	23 (38.9)
PD = periodontal disease	*N* = 59

## Data Availability

The data used to support the findings of this study are included within the article.
